# Theory-based correlates of cannabis use and intentions among US and Israeli adults: a mixed methods study

**DOI:** 10.1186/s13011-023-00562-x

**Published:** 2023-09-06

**Authors:** Yuxian Cui, Cassidy R. LoParco, Yael Bar-Zeev, Zongshuan Duan, Hagai Levine, Lorien C. Abroms, Yan Wang, Amal Khayat, Carla J. Berg

**Affiliations:** 1 Department of Prevention and Community Health, Milken Institute School of Public Health, George Washington University, Washington, DC USA; 2 Braun School of Public Health and Community Medicine, Faculty of Medicine, The Hebrew University of Jerusalem and Hadassah Medical Center, Jerusalem, Israel; 3 Department of Population Health Sciences, School of Public Health, Georgia State University, Atlanta, GA USA

**Keywords:** Cannabis use, Global health, Theory, Health policy, Non-medical cannabis, Recreational cannabis, Health behavior

## Abstract

**Background:**

In the US and Israel, non-medical (‘recreational’) cannabis use is illegal at the national level; however, use rates are high and decriminalization and legalization is spreading. Thus, theory-based intervention efforts, especially for youth prevention, are crucial.

**Methods:**

This mixed-methods study of adults in the US (*n* = 1,128) and Israel (*n* = 1,094) analyzed: 1) cross-sectional survey data (Fall 2021) to identify theory-based correlates (risk perceptions, social norms) of past-month cannabis use, next-year use intentions, and intentions to use in the home or among children if non-medical cannabis was legal, using multivariable regression; and 2) qualitative interviews regarding perceptions of cannabis policies and use (US *n* = 40, Israel *n* = 44).

**Results:**

16.7% reported past-month use; 70.5%, 56.3%, and 82.6% indicated “not at all likely” regarding next-year use and use in the home and among children if legal. Lower perceived risk and greater social norms were associated with past-month use, greater use intentions, and greater intentions to use in the home or among children. Past-month use was more prevalent among US (vs. Israeli) participants (22.0% vs. 11.2%); however, in multivariable regression controlling for past-month use, being from Israel was associated with greater use intentions (next-year; in the home/among children). Qualitative themes indicated: concerns about use (e.g., increasing use, health risks, driving-related risks) and legalization (e.g., impact on society/economy, marketing), and perceived benefits of use (e.g., medical) and legalization (e.g., access/safety, economic, individual rights).

**Conclusions:**

Despite differences in cannabis perceptions and use across countries, perceived risk and social norms are relevant intervention targets regardless of sociopolitical context.

## Background

Cannabis use is an increasing global health concern [1]. Although cannabis use may effectively address certain medical conditions [2], it may have negative health effects (e.g., immune function [3], respiratory function [4], mental health [5]) and socioeconomic (e.g., academic, employment) outcomes [6–8] and disproportionately impacts certain subpopulations (e.g., men, sexual minorities, racial/ethnic minorities) [9–13].

Despite these concerns, the global cannabis market has dramatically grown [14]. North American accounts for 96.8% ($22 billion) of the global legal cannabis market, with the US accounting for $20 billion (Canada $2 billion). Israel accounts for ~ 22% of the remainder of the market (second only to Germany, ~ 28%) [14]. Moreover, the US and Israel represent countries with the highest proportion of adults who consume cannabis [14]. Per 2020 data, 17.4% of US adults and 27.0% of Israeli adults reported past-year cannabis use [14]. Cannabis legislation in both countries has markedly changed over the past decades. As of November 2022, 38 states in the US have legalized medical cannabis [15]. Additionally, 21 states and the District of Columbia legalized non-medical (‘recreational’) cannabis use for adults (≥ 21 years-old) [15], the first of which date back to 2012 (Colorado, Washington) [15]. In Israel, in 2011, the Israel Medical Cannabis Agency was established to regulate medical cannabis [16], and non-medical cannabis use for adults (≥ 18 years-old) was decriminalized in 2019 [17, 18]. In 2020, 2 bills to allow non-medical cannabis sales passed preliminary readings in Israel’s parliament [19]. In 2021, another bill to legalize non-medical cannabis use was approved by the Ministerial Committee for Legislation, but rejected by parliament [20].

As legislative contexts have shifted in these 2 distinct countries with high cannabis use rates, cannabis-related perceptions and use-related behaviors are likely to change [21–27]. For example, cannabis legalization may be associated with more favorable perceptions (e.g., lower perceived risk, greater social norms) and increased use intentions among young adults [21], higher use prevalence and levels among youth [22, 28], greater use among adults with children living in the home [29], and changes in use motives (e.g., recreational vs. medical), modes of use, and product source among those using cannabis [13, 28, 30–32]. However, the evidence is mixed [33].

Understanding how adults address cannabis use within their homes or around children is critical in mitigating use-related risks among both adults and young people. One important consideration is the restrictiveness of home environments. For example, the literature regarding tobacco suggests that allowing use in the home is associated with greater secondhand smoke exposure and cigarette consumption, while prohibiting use is associated with lower consumption, more attempts to quit use, and higher quit rates [34]. Notably, children who live in homes that allow smoking are more likely to initiate smoking themselves [34]. Additionally, the literature underscores the importance of parenting, including parental substance use and monitoring, in influencing youth substance use [35, 36], including cannabis [37]. This is important given the literature indicating increased cannabis use among adults with children in the home post-legalization [29] and increased use among youth and young adults [21, 22, 28, 38].

Social Cognitive Theory (SCT) [39] highlights the dynamic interplay of one’s social context (e.g., social roles, social norms) and cognitions such as outcome expectancies (e.g., perceived risks or consequences vs. benefits) in relation to one’s behaviors. For example, an individual’s perceptions of how socially acceptable or normative cannabis use is likely depends on the nature and extent of their exposure to use within their social networks, and these perceptions ultimately impact their personal use [9–12, 37, 40–42]. Additionally, the expected outcomes of use – either positive or negative – influence whether one uses cannabis; if one expects positive outcomes, like a pleasurably psychological experience, they are more likely to use, while if one expects negative outcomes, like health risks or addiction, they may be less likely to use [9–12, 37, 40–42]. Moreover, if one perceives negative implications of others (e.g., family members or children in the home) being exposed to cannabis use (e.g., health risks, enticing youth), they may be more likely to implement restrictions about cannabis use in the home or in the presence of children. Indeed, in the tobacco literature, SCT has proven to be a useful framework for understanding the implementation of smoke-free homes to protect children and nonsmokers [43–47].

While SCT provides a useful model for identifying factors associated with substance use related outcomes and cannabis use outcomes [9–12, 37, 40–42], existing literature regarding how adults address cannabis use within their homes or in the presence of children is limited. Furthermore, little cross-country research has examined theory-based constructs related to cannabis-related outcomes in differing sociopolitical contexts.

To advance the literature and inform regulatory and prevention efforts, this mixed-methods study identified: 1) theory-based correlates (risk perceptions, social norms) of past-month cannabis use, next-year use intentions, and intentions to use in the home or near children if non-medical cannabis was legal among US and Israeli adults; and 2) qualitative themes regarding perceptions of cannabis use and policies.

## Methods

We analyzed data from a study of US and Israeli adults that used a sequential explanatory mixed-methods design and primarily focused on tobacco-related perceptions and use [48]. Eligibility criteria included: 1) age 18–45 years; and 2) able to speak English (US), or Hebrew or Arabic (Israel); in Israel, an additional criterion was having an Israeli ID. The study received ethical approvals from George Washington University (NCR213416) and Hebrew University (27062021). The current study analyzed: 1) cross-sectional survey data (collected in October-December 2021); and 2) semi-structured interviews (conducted in Spring 2022). This study adhered to STROBE guidelines for cross-sectional quantitative research and COREQ guidelines for qualitative research.

### Quantitative Data

#### Participants

The US survey was conducted primarily using KnowledgePanel®, a probability-based web panel designed to be representative, recruited via random-digit dialing and address-based sampling, and incentivized via KnowledgePanel® points redeemable for cash. This approach was supplemented with off-panel participant recruitment (via banner ads, web pages) to meet subgroup recruitment targets (i.e., Asian individuals reporting tobacco use). Of 4,960 panelists recruited, 2,397 (48.3%) completed eligibility screening, and 1,095 (45.7%) completed the survey; of 353 off-panel individuals screened, 33 (9.3%) were eligible and completed the survey. The Israeli survey was conducted using opt-in sampling, as described above. Of 2,970 individuals screened and eligible, 1,094 (36.8%) completed the survey.

#### Measures

The survey took ~ 25 min to complete and was professionally translated to Hebrew and Arabic for Israeli participants.

##### Outcomes: Cannabis use, use intentions, and intentions to allow use in the home and/or near children if legalized

Participants were asked, “How old were you when you first used marijuana?” including the option of “I have never used marijuana” [49]. Those reporting lifetime use were asked, “During the past 30 days, on how many days did you use marijuana?” [49]. Among all participants, we assessed cannabis use intentions by asking, “How likely are you to try or continue to use marijuana in the next year?” (1 = not at all to 7 = extremely). We also asked, “If marijuana were legalized for recreational use, how likely would you be to allow marijuana use: in your home? in the presence of children?” (1 = not at all to 4 = very); responses to these 2 items were averaged to create an index score.

##### Cannabis use characteristics

Participants reporting lifetime use were asked, “How have you used marijuana in the last 12 months?” [50]. Response options (e.g., vaped in liquid form) are shown in Table 1. Those reporting past 30-day (current) use were asked, “Which is the one method you used most in the last 12 months?” with the same response options [50]. Those reporting lifetime use were also asked, “For which of the following reasons do you primarily use marijuana? recreational purposes; medical purposes; or both recreational and medical purposes” and “In the last 12 months, where have you most often obtained marijuana?” (response options in Table 1) [50]. Those reporting current use also reported times used per day on days used.

**Table 1:** Participant characteristics and cannabis-related factors among US and Israeli participants

		**Country**		
	**Overall**	**US**	**Israel**	
	***N* = 2,222**	***N* = 1,128**	***N* = 1,094**	
	**(100%)**	**(50.8%)**	**(49.2%)**	
**Variables**	**N (%) or** **M (SD)**	**N (%) or** **M (SD)**	**N (%) or** **M (SD)**	***p***
***Sociodemographics***	–	–	–	–
Age, M (SD)	32.19 (7.74)	34.11 (7.23)	30.21 (7.76)	** < .001**
Female, N (%)	1,118 (50.3)	562 (49.8)	556 (50.8)	.637
Sexual minority, N (%)	356 (16.0)	147 (13.0)	209 (19.1)	< .001
≥ College degree, N (%)	953 (42.9)	484 (42.9)	469 (42.9)	.986
Married/cohabitating, N (%)	1,186 (53.4)	601 (53.3)	585 (53.5)	.927
Children in the home, N (%)	1,125 (50.6)	529 (46.9)	596 (54.5)	** < .001**
***Theory-based predictors, M (SD)***				
*Perceived risk – mean score *^*a*^	4.75 (1.97)	4.29 (1.99)	5.18 (1.84)	** < .001**
Addictiveness	4.76 (2.25)	4.40 (2.26)	5.12 (2.19)	** < .001**
Harm to health	4.73 (2.20)	4.20 (2.22)	5.25 (2.05)	** < .001**
*Perceived social norms – mean score *^*b*^	2.49 (1.60)	2.79 (1.70)	2.19 (1.44)	** < .001**
Social acceptability	2.58 (1.90)	2.93 (2.04)	2.24 (1.69)	** < .001**
Use in social network	2.40 (1.68)	2.64 (1.70)	2.15 (1.61)	** < .001**
	***N* = 809**	***N* = 563**	***N* = 246**	
**Those reporting lifetime cannabis use:**	**(36.4%)**	**(49.9%)**	**(22.5%)**	** < .001**
Age of first use, M (SD)	18.28 (5.83)	17.83 (5.22)	19.31 (6.95)	**.001**
Methods used, past 12 months, N (%)				
Smoked without tobacco	244 (30.2)	202 (36.0)	42 (17.2)	** < .001**
Smoked with tobacco	167 (20.7)	48 (8.6)	119 (48.4)	** < .001**
Vaped in liquid form	140 (17.3)	109 (19.4)	31 (12.6)	**.018**
Vaped dried leaves or herb	59 (7.3)	34 (6.1)	25 (10.2)	**.039**
Dabbed concentrates (e.g., shatter, budder, wax)	59 (7.3)	50 (8.9)	9 (3.7)	**.008**
Orally (e.g., oil, capsules, dissolvable strips, spray)	51 (6.3)	38 (6.8)	13 (5.3)	.424
Topically (e.g., lotions, bath salts)	39 (4.8)	28 (5.0)	11 (4.5)	.751
Edibles (i.e., food/drinks)	178 (22.1)	152 (27.1)	26 (10.6)	** < .001**
Primary reasons for cannabis use, N (%)^c^				**.015**
Recreational purposes	479 (66.3)	345 (68.5)	134 (61.2)	
Medical purposes	66 (9.1)	36 (7.1)	30 (13.7)	
Both recreational and medical purposes	178 (24.6)	123 (24.4)	55 (25.1)	
Source of cannabis, most often in past 12 months, N (%)^d^				** < .001**
Family member or friend	184 (41.7)	119 (41.5)	65 (42.2)	
Dealer or other non-legal source (in person)	91 (20.6)	52 (18.1)	39 (25.3)	
At vape shop, dispensary, co-operative, or other store	100 (22.7)	91 (31.7)	9 (5.8)	
Online	66 (15.0)	25 (8.7)	41 (26.6)	
	***N* = 370**	***N* = 248**	***N* = 122**	
**Those reporting past 30-day cannabis use:**	**(16.7%)**	**(22.0%)**	**(11.2%)**	** < .001**
Number of days used, past 30 days, M (SD)	13.84 (11.85)	15.90 (12.29)	9.66 (9.67)	** < .001**
Times used per day on days used, M (SD)	3.29 (2.89)	3.26 (3.00)	3.37 (2.69)	.728
Most common method, past 12 months, N (%)				** < .001**
Smoked without tobacco	123 (34.3)	113 (47.3)	10 (8.3)	
Smoked with tobacco	94 (26.2)	25 (10.5)	69 (57.5)	
Vaped	75 (20.9)	47 (19.7)	28 (23.3)	
Edibles (i.e., food/drinks)	28 (7.8)	27 (11.3)	1 (0.8)	
Other	39 (10.9)	27 (11.3)	12 (10.0)	

Correlation among items: ^a^ r = .41. ^b^ r = .53

^c^Don’t know, *n* = 43; Prefer not to answer, *n* = 39

^d^Other, *n* = 33; Don’t know, *n* = 15; None of the above/didn’t use or obtain, *n* = 266; Prefer not to answer, *n* = 53

##### Perceived risk and social norms

To assess SCT-related social-cognitive constructs related to outcome expectancies [39], we assessed perceptions of risk and social norms. To assess perceived risk, participants were asked, “How [addictive; harmful to your health] do you think marijuana is?” (1 = not at all to 7 = extremely) [51]; responses were averaged to create an index score. To assess social norms, participants were asked, “Please indicate the extent to which people important to you would (or do) approve of you using marijuana?” (1 = all disapprove to 7 = all approve) and “How many of your closest connections (including your partner, friends, relatives, co-workers, and others) use marijuana?” (1 = none to 7 = all) [51]; responses were averaged to create an index score.

##### Sociodemographic covariates

We included country, age, sex, education, marital status, and children in the home. (Note: Given differences in state cannabis legalization in the US, participants were also coded as residing in a state with legal non-medical cannabis versus not to explore this factor in US-specific preliminary analyses.)

#### Data analysis

First, descriptive and bivariate analyses (Chi-square for categorical variables, t-tests or ANOVAs for continuous variables) characterized participants across countries with regard to sociodemographics and cannabis use characteristics and outcomes. Next, bivariate analyses assessed correlates of interest in relation to cannabis use, use intentions, and intentions to allow use in the home/near children if legalized, separately. Then, multivariable regression analyses examined correlates of these 3 outcomes (binary logistic regression for current use, linear regressions for use intentions and intentions to allow use in the home/near children if legalized). Country, sociodemographics, and perceived risk and social norms were included in the models; current cannabis use was also included in the models predicting use intentions and intentions to allow use in the home/near children if legalized.

Exploratory analyses also assessed country-specific models, which yielded similar findings to the overall models; further, no significant interactions between country and correlates of interest were found in relation to any outcome. We also examined US state non-medical cannabis legalization in relation to cannabis use characteristics and outcomes among US participants. Those in legalized states reported greater social norms, lifetime use, and legal sources; no other differences were found, and state legalization were not significantly associated with cannabis-related outcomes in US-specific models. Thus, we presented the overall models, including country as a covariate. Quantitative analyses were conducted by SPSS (26.0), using alpha = 0.05.

### Qualitative data

#### Participants

Participants in both countries were purposively recruited for representation by sex and race/ethnicity. In the US, participants were recruited from the online survey sample and were called and/or emailed an invitation to participate. In Israel, the opt-in sample for the online survey precluded our ability to re-contact survey participants; instead, we promoted the study via ads on Facebook; potential participants were provided a study description, consented, and screened for eligibility (i.e., ≥ 18 years old, speak Hebrew or Arabic).

#### Assessment

Interviews were guided by standard principles of qualitative methods. [52, 53] Each interview was conducted online via Zoom in English (US) or Hebrew/Arabic (Israel; participant’s choice), audio-recorded, ~ 45 min long, and incentivized (USD$25 or 100 NIS). The interview guide included various questions about cannabis and tobacco use. Questions relevant to the current study assessed perceptions of cannabis use (e.g., “If marijuana were legalized for recreational use, how likely would you be to allow marijuana use in your home? Why or why not?”) and cannabis policies (e.g., “What do you think about recreational marijuana policies?”). Interviews were transcribed by a professional transcription service.

#### Qualitative analysis

Qualitative data were analyzed using standard principles of qualitative methods [52–54] and deductive-inductive thematic analysis. [55] A preliminary set of deductive codes were compiled based on the interview guide and a preliminary review of the US-based transcripts. Then, a subsample of 8 US transcripts and 8 translated Arabic and Hebrew interviews (n = 4 each) were independently reviewed by 2 US-based coders and 2 Israeli-based coders (1 from each team per transcript). An iterative process was used to assess inter-rater reliability, reach consensus, inform revisions, and yield additional codes based on emergent themes. [55] These codes were compiled into a codebook. After ensuring sufficient inter-rater reliability (> 80%), the remainder of the interviews were coded. Representative quotes were selected for inclusion in the manuscript.

## Results

### Quantitative results

#### Participant characteristics

In this sample (*N* = 2,222; US *n* = 1,128, Israel *n* = 10.94), participants were an average age of 32.19 (SD = 7.74), and 50.3% were female; further, 36.4% reported lifetime cannabis use and 16.7% current use (Table 1). The average score for next-year use intentions was 2.07 (SD = 1.95, scale: 1–7), with 70.5% indicating “not at all likely”. Average scores for intentions to use (if legalized) in the home and in the presence of children were 1.87 (SD = 1.15, scale: 1–4) and 1.30 (SD = 0.73), with 56.3% and 82.6% indicating “not at all likely”, respectively.

Bivariate results characterizing factors associated with current use, use intentions, and intentions to allow use in the home or near children if legal are shown in Table 2. Participants residing in the US (vs. Israel) more likely reported lifetime (49.9% vs. 22.5%) and current use (22.0% vs. 11.2%) and reported lower risk perceptions and greater social norms (*p*’s < 0.001).

**Table 2:** Bivariate analyses examining participant characteristics in relation to past 30-day cannabis use, intentions to use, and intentions to allow use in the home or near children if legalized among US and Israeli adults, *N* = 2,222

	**Total** ***N* = 2,222** **(100%)**	**Cannabis use**			**Intentions to use cannabis in the next year**		**Intentions to allow use in home/near children if legal**^a^	
		**No** ***N* = 1,852 (83.3%)**	**Yes** ***N* = 370 (16.7%)**					
	**N (%) or**	**N (%) or**	**N (%) or**		**M (SD)**		**M (SD)**	
**Among all participants:**	**M (SD)**	**M (SD)**	**M (SD)**	***p***	**or r**	***p***	**or r**	***p***
***Sociodemographics***								
Country, N (%) and M (SD)				** < .001**		** < .001**		.977
US	1,128 (50.8)	880 (47.5)	248 (67.0)		2.30 (2.17)		1.58 (0.80)	
Israel	1,094 (49.2)	972 (52.5)	122 (33.0)		1.85 (1.68)		1.58 (0.79)	
Age, M (SD) and r	32.19 (7.74)	32.06 (7.79)	32.82 (7.50)	.083	.04	.081	-.001	.958
Gender, N (%) and M (SD)				**.022**		**.002**		**.004**
Male	1,104 (49.7)	900 (48.6)	204 (55.1)		2.21 (2.02)		1.63 (0.82)	
Female	1,118 (50.3)	952 (51.4)	166 (44.9)		1.95 (1.88)		1.53 (0.77)	
Sexual orientation, N (%) and M (SD)				**.001**		**.019**		.093
Heterosexual/straight	1,866 (84.0)	1,576 (85.1)	290 (78.4)		2.03 (1.91)		1.57 (0.79)	
Sexual minority	356 (16.0)	276 (14.9)	80 (21.6)		2.30 (2.14)		1.65 (0.81)	
Education, N (%) and M (SD)				**.013**		.077		.720
< College degree	1,269 (57.1)	1,036 (55.9)	233 (63.0)		2.14 (2.03)		1.58 (0.79)	
≥ College degree	953 (42.9)	816 (44.1)	137 (37.0)		1.99 (1.84)		1.59 (0.80)	
Marital status, N (%) and M (SD)				.154		.639		.753
Married/cohabitating	1,186 (53.4)	1001 (54.0)	185 (50.0)		2.06 (1.95)		1.58 (0.80)	
Other	1,036 (46.6)	851 (46.0)	185 (50.0)		2.10 (1.95)		1.59 (0.79)	
Children in the home, N (%) and M (SD)				** < .001**		.**015**		**.001**
No	1,097 (49.4)	881 (47.6)	216 (58.4)		2.18 (2.00)		1.64 (0.81)	
Yes	1,125 (50.6)	971 (52.4)	154 (41.6)		1.97 (1.90)		1.53 (0.79)	
***Theory-based predictors,*** M (SD) and r								
*Perceived risk – mean score*	4.75 (1.97)	4.97 (1.94)	3.62 (1.72)	** < .001**	-.28	** < .001**	-.34	** < .001**
Addictiveness	4.76 (2.25)	4.93 (2.25)	3.91 (2.05)	** < .001**	-.18	** < .001**	-.24	** < .001**
Harm to health	4.73 (2.20)	5.01 (2.14)	3.34 (1.95)	** < .001**	-.32	** < .001**	-.36	** < .001**
*Perceived social norms – mean score*	2.49 (1.60)	2.11 (1.33)	4.37 (1.49)	** < .001**	.64	** < .001**	.56	** < .001**
Social acceptability	2.58 (1.90)	2.20 (1.67)	4.46 (1.88)	** < .001**	.55	** < .001**	.51	** < .001**
Use in social network	2.40 (1.68)	2.02 (1.39)	4.28 (1.69)	** < .001**	.60	** < .001**	.50	** < .001**
***Past 30-day cannabis use***, N (%) and M (SD)				**–**		** < .001**		** < .001**
No	1852 (83.3)	**–**	**–**		1.44 (1.20)		1.42 (0.70)	
Yes	370 (16.7)	**–**	**–**		5.25 (1.91)		2.41 (0.75)	

^a^Index score calculated as average of 2 items: “If marijuana were legalized for recreational use, how likely would you be to allow marijuana use: in your home? in the presence of children?” (1 = not at all to 4 = very); correlation among items: r = .42

#### Cannabis use and related characteristics

In multivariable regression analyses (Table 3), correlates of current cannabis use included lower perceived risk (aOR = 0.80, 95%CI = 0.74, 0.87) and greater norms (aOR = 2.39, 95%CI = 2.17, 2.63), as well as being male (aOR_female_ = 0.6, 95%CI = 0.52, 0.93) and sexual minority (aOR = 1.67, 95%CI = 1.15, 2.40; *p*’s < 0.05; Nagelkerke R-square = 0.432). Bivariate analyses (Table 1) indicated that, among participants reporting lifetime use, US (vs. Israeli) participants reported younger first age of use, more likely using via smoking without tobacco, vaping in liquid form, dabbing/concentrates, and edibles, but less likely via smoking with tobacco or vaping dried leaves or herbs (*p*’s < 0.05). US participants reporting lifetime use were more likely using primarily recreationally and obtaining it from retailers (*p*’s < 0.05). Among those reporting current use, US participants reported more days of use (*p* < 0.001).

**Table 3:** Multivariable regression models examining correlates of past 30-day cannabis use, intentions to use cannabis, and intentions to allow use in the home or near children if legalized among US and Israeli adults, *N* = 2,222

	**Cannabis use**			**Intentions to use** **cannabis in the** **next year**				**Intentions to allow use** **in home/near children** **if legal**^c^			
**Variables**	**aOR**	**95%CI**	***p***	**Beta**	**B**	**95%CI of B**	***p***	**Beta**	**B**	**95%CI of B**	***p***
***Sociodemographics***											
Israel (ref: US)	0.84	0.62, 1.14	.258	0.03	0.13	0.02, 0.24	**.019**	0.14	0.23	0.17, 0.29	** < .001**
Age	1.01	0.99, 1.03	.304	0.01	0.001	-0.01, 0.01	.751	-0.01	-0.001	-0.01, 0.00	.580
Female (ref: male)	0.69	0.52, 0.93	.**013**	-0.04	-0.17	-0.27, -0.06	.**002**	-0.40	-0.06	-0.12, -0.01	**.027**
Sexual minority (ref: heterosexual)	1.67	1.15, 2.40	**.006**	0.01	0.03	-0.11, 0.17	.674	0.01	0.02	-0.06, 0.10	.600
≥ College degree (ref: < college degree)	0.78	0.58, 1.05	.101	0.01	0.03	-0.08, 0.14	.555	0.04	0.06	0.001, 0.12	**.038**
Married/cohabitating (ref: other)	0.80	0.57, 1.11	.175	-0.01	-0.05	-0.17, 0.07	.382	-0.01	-0.01	-0.08, 0.05	.655
Children in the home (ref: no)	0.78	0.57, 1.06	.111	0.03	0.10	-0.02, 0.21	.092	-0.01	-0.01	-0.07, 0.05	.650
***Theory-based predictors***											
Perceived risk	0.80	0.74, 0.87	** < .001**	0.54	-0.04	-0.07, -0.01	.**006**	0.21	-0.07	-0.09, -0.06	** < .001**
Perceived social norms	2.39	2.17, 2.63	** < .001**	-0.04	0.43	0.39, 0.47	** < .001**	-0.18	0.21	0.19, 0.23	** < .001**
***Past 30-day cannabis use*** (ref: no)	-	-	-	0.36	2.80	2.63, 2.96	** < .001**	0.42	0.45	0.37, 0.54	** < .001**
*R-square*		.432 ^a^		.629 ^b^				.391 ^b^			

Beta: Standardized coefficient. B: Unstandardized coefficient

^a^Nagelkerke R-square

^b^Adjusted R-square

^c^Index score calculated as average of 2 items: “If marijuana were legalized for recreational use, how likely would you be to allow marijuana use: in your home? in the presence of children?” (1 = not at all to 4 = very)

#### Cannabis use intentions

In multivariable regression (Table 3), correlates of greater use intentions included lower perceived risk (B = -0.04, 95%CI = -0.07, -0.01), greater norms (B = 0.43, 95%CI = 0.39, 0.47), and current cannabis use (B = 2.80, 95%CI = 2.63, 2.96), as well as being from Israel (B = 0.13, 95%CI = 0.02, 0.24) and male (B_female_ = -0.17, 95%CI =—0.27, -0.06; p’s < 0.05; Adjusted R-square = 0.629).

#### Intentions to allow use in the home and near children if legalized

In multivariable regression analysis (Table 3), correlates of intentions to allow use in the home or near children if legal included lower perceived risk (B = -0.07, 95%CI = -0.09, -0.06), greater norms (B = 0.21, 95%CI = 0.19, 0.23), and current use (B = 0.45, 95%CI = 0.37, 0.54), as well as being from Israel (B = 0.23, 95%CI = 0.17, 0.29), male (B_female_ = -0.06, 95%CI = -0.12, -0.01), and more educated (B = 0.06, 95%CI = 0.001, 0.12; *p*’s < 0.05; Adjusted R-square = 0.391).

### Qualitative results

US participants were 36.5 (SD = 6.3) years-old, 42.5% female, 32.5% non-Hispanic (NH) White, 32.5% NH Black, 12.5% NH Asian, and 22.5% Hispanic; 52.5% reported current cannabis use. Israeli participants were on average 29.35 (SD = 6.2) years old, 52.3% female, 88.6% Jewish, and 11.4% Arab. Several themes emerged with regard to perceived risks and benefits of cannabis use and non-medical cannabis legalization (Table 4).

**Table 4:** Qualitative findings regarding perceptions of cannabis use and non-medical legalization among US and Israeli adults

**Themes**	**Representative Quotes**
**Perceptions of cannabis use**	
***No concerns***	‒ Children can be aware of what is happening in the world and around them. There is no need to withhold from them or conceal such information from them. (38 year-old Jewish female, Israel)
***Perceived risks***	
Social normalization for children	‒ Children are very impressionable. Smoking or using marijuana or getting high is not something that kids need to see and take in as a good or normal thing. I don’t think that [cannabis use] should be allowed in front of kids. (30 year-old NH White male, US) ‒ Even if it’s legal, there’s still some boundaries or respect that you still should have. I would not do that in front of others and in front of children. (24 year-old Hispanic male, US)
Impact of byproducts on health of children	‒ I would not allow [cannabis use] in the presence of children just because I don’t think smoking, even if it's marijuana, is healthy for children. And I don’t think it should be around children. They can’t consent. (39 year-old NH Asian female, US) ‒ I would permit this from above a certain age, like 12, 13 or something, mainly because of the smoke coming out. It’s a health thing. When they’re small, they’re simply more vulnerable. I would permit it because at least a child above age 13 [can voice and take actions for themselves]. I can speak for myself as someone whose parents smoke. If the smoke would bother me, I’d move away from the smoke. (21 year-old Jewish male, Israel)
Risks associated with use among young people	‒ I know some people that are really sort of addicted to marijuana, and that is what bothers me. And of course, use by young people is very important to me, even age 24, which is the age at which the front of the brain develops the fastest, and marijuana also has an effect on the brain, and it can harm all sorts of characteristics that we acquire during our lives. (24 year-old Jewish female, Israel)
Cannabis serving as a gateway drug	‒ I think that [cannabis] is definitely a gateway drug. I think that most people, especially younger people, don't simply just stop at smoking marijuana. (30 year-old NH White male, US)
Health risks of cannabis use	‒ [Cannabis use] is no different than drinking alcohol or smoking cigarettes. All these things are harmful, all these things have effects, both emotional and cognitive. (38 year-old Jewish female, Israel) ‒ I never had any interaction or, you know, exposure to [cannabis]. But to my knowledge, [cannabis] is extremely dangerous, because they may drive people crazy, you know, and the harmful effects are not clearly known. (40 year-old NH Asian male, US)
Driving under the influence of cannabis	‒ I definitely hate the idea of people smoking a bunch of weed and then driving. That is incredibly dangerous. (44 year-old, NH White female, US) ‒ I would probably be more scared of weed because you have your legal limit of alcohol. You can go out and have dinner, and usually you’re safe to have at least like one drink and then drive. But, is there a limit on how much you're safe to drive with weed? I wouldn't trust myself to have a tiny bit of a THC gummy and get behind the wheel. (39 year-old NH White female, US)
***Perceived benefits***	
Medical benefits of cannabis	‒ I think marijuana has a lot of medical uses and can be used to help a lot of people. (32 year-old NH White female, US) ‒ [Cannabis] helps with pain, with sleeping, so I'm for recreational marijuana. (39 year-old NH Black male, US) ‒ I enjoy [cannabis] myself. I have chronic pain and anxiety, and it does help me. I know it helps a lot of people with chronic problems. (39 year-old NH White female, US) ‒ Marijuana has many advantages. It also has many medical advantages. It could be a mental condition or some sort of anxiety attack or things that are happen in our day-to-day lives. (24 year-old Jewish female, Israel)
Not as dangerous as opioids	‒ I think [cannabis] is a far better alternative than the big opioid thing we have going on with people addicted to pills and stuff like that. I feel like marijuana is a far better, healthier option because it is a plant. (23 year-old NH White female, US) ‒ Peer reviewed evidence suggested that cannabis have better outcomes than traditional painkillers, which are very costly and harm your organs versus cannabis, unless you do it a little too much. (27 year-old Hispanic male, US)
Not as dangerous as alcohol	‒ In my personal opinion, I think [cannabis] does affect you a little bit, but not at all to the extent of like alcohol or anything like that. (33 year-old NH White female, US) ‒ [Cannabis] affects everyone differently, but I think it affects the driver’s alertness. I mean the person, I won’t say they’re sober, but cannabis is not alcohol. Alcohol takes it to the extreme. Cannabis can simply slow you down. Again, it can also be harmful, causing a lack of vigilance. ([unknown age] Jewish female, Israel)
Not as dangerous as tobacco	‒ I would authorize [cannabis] over cigarettes, just because I would find that there's less harmful ingredients or chemicals in the [cannabis] product versus traditional cigarettes. It just depends on how often or how frequently the product is being used. But I would just associate with it being a more natural product versus cigarettes. (27 year-old Hispanic male, US)
**Perceptions of non-medical (i.e., ‘recreational’) cannabis legalization**	
***No concerns***	‒ As long as [cannabis] is well regulated, I don’t have concerns. (39 year-old NH Asian female, US) ‒ I don’t see a problem with [legalizing non-medical cannabis]. I personally love it. To be honest, I don’t think it's even something that needs to be regulated by the government. (32 year-old NH Black female, US) ‒ I have no problem with [legalizing non-medical cannabis]. [Cannabis] is no different than alcohol. In my opinion, I don’t see why [cannabis] should be restricted, particularly if alcohol, let’s say, isn’t controlled. (38 year-old Jewish female, Israel) ‒ For a certain age group, I wouldn’t be against [legalizing non-medical]. I don’t think it’s harmful. (35 year-old Arab female, Israel)
***Perceived risks***	
Negative impact on society	‒ Laws should be very strict. Definitely, such policies would really help saving people falling in trap of these products. And if any small leverage of these [policies] occurred under the pretext of making some exemptions, it would really have a harmful effect to the society's families and communities. (40 year-old NH Asian male, US)
Negative impact on economy and productivity	‒ I guess [cannabis] creates a society that tends to be less productive in a time where we need more people to be productive. We need more workforce, we have millions of jobs that are not being used, and you can't hire people because of drug use. So, we have a huge drug problem in this country. And I don't think that making marijuana more open and legal helps that. (30 year-old NH White male, US)
Increased crime	‒ There has been a huge uptick of robberies of these pot stores. It's scary. People coming in with guns and stuff. (39 year-old NH White female, US)
Inequitable economic benefit	‒ People who’ve been selling weed for forever and getting in trouble for it are going to be left out of the opportunity to make money now when it's legal. (37 year-old NH Black female, US) ‒ I think that they’re going to legalize [non-medical cannabis], and people who shouldn't be making money off of it are going to make money off of it. (37 year-old NH Black female, US)
Increased price of cannabis products	‒ [Legalizing non-medical cannabis] will probably also increase the price. Everyone suddenly wants a share, so everyone will want a share until it reaches the store. ([unknown age] Jewish female, Israel)
Cannabis marketing	‒ It's weird when a piece of marijuana looks like brownie, candy or like gummy bears. I disagree with that. It's not like I ever bought it or tried it, or I will ever do it. Never. But still, for me, there should be more regulations. (45 year-old NH Asian female, US) ‒ What’s bad is that, at least today, most cannabis advertising says it’s healthy. I think this is unwise because it encourages people to use, and I don’t think there’s enough knowledge available to be able to say what’s more or less healthy. I’d be pleased, for instance, if they would at least require warnings that say that this hasn’t been studied enough and there may be side effects and things. (21 year-old Jewish male, Israel)
Insufficient regulation	‒ My concerns would just be related to lack of regulation of the products – so potential contaminants and lack of oversight in facilities where the products are processed and packaged. (38 year-old NH White female, US) ‒ I have concerns over the controls, like quality control. And there still seems to be a pretty significant black market for marijuana products. I think there’s still a lot of room for, like fly by night companies, to produce products. (39 year-old NH Black male, US)
Driving under the influence	‒ The only concern I have is that if it were federally legal. I feel like it definitely does impact your thinking and your ability to drive and things like that. I think that would be a concern. (23 year-old NH White female, US) ‒ There should also be a prohibition on driving under the influence of marijuana just like [the government did] with alcohol. Marijuana should be treated like alcohol. (38 year-old Jewish female, Israel)
***Perceived benefits***	
Decriminalization	‒ [Legalizing non-medical cannabis] makes it less criminal. You're not going to jail anymore, or getting fined or arrested for having it on you. I think that's a good thing. I think it's a waste of resources to punish people for having it. (39 year-old NH Asian female, US) ‒ I support legalization for recreational use. I think it would stop crime in some urban areas and will cut down on the overt incarceration of African American and brown people, and it’ll help underserved communities recoup lost funds. (37 year-old NH Black female, US) ‒ I keep comparing [cannabis] to alcohol. Alcohol’s legal, like people celebrate with alcohol. So it just doesn't make sense that one is legal and one is super illegal. Federally, I think it's still a schedule one drug or whatever. That’s just nuts to me. (27 year-old Hispanic male, US) ‒ If you make something already happening legal, I assume this reduces criminality around the whole business. (38 year-old Jewish female, Israel)
Facilitating medical cannabis use	‒ I think a lot of people might be able to get a lot of medicine for the medical needs, when they can't get it right now. Like a lot of kids need it for seizures and stuff like that. (42 year-old NH Black male, US) ‒ I know the government kind of thinks a little bit otherwise, but I think [legalizing non-medical cannabis] would help a lot of disabled people and people that have anxiety, you know, help them with their diseases. (32 year-old NH White female, US)
Increased accessibility of cannabis products	‒ [Legalizing non-medical cannabis] makes [cannabis] accessible, so you don't have to go to sketchy places to get products. You can go to an easily-accessed store. (39 year-old NH Asian female, US) ‒ [Legalizing cannabis] is good, then if I buy marijuana, all the products I need to use marijuana should be accessible. (24 year-old Jewish female, Israel)
Increased safety and regulation	‒ I think it's good that you're able to go into the store and purchase [cannabis] versus buying it on the streets, not knowing what that person has done to it. Because what I'm hearing is that it could be laced with fentanyl, and people are actually dying. I feel more comfortable with something that is approved by the government to be in a store vs. buying something on the streets. (44 year-old NH Black female, US) ‒ People wouldn’t have to go somewhere where someone could have done something to the product that could be harmful. You’d have a safe place where you can go get something legally, and feel safe. (24 year-old Hispanic male, US) ‒ Legalizing [non-medical cannabis] would allow more control versus it being sold in the shadows. So I feel like it's a good thing to be legalized and actually have more control over what's happening. (28 year-old NH Black female, US) ‒ The point is that this legal audit has advantages and disadvantages. So the advantages are that it is clean, and it undergoes an audit and not everyone can sell whatever junk they want. ([unknown age] Jewish female, Israel) ‒ Cannabis use is already happening. And when [non-medical cannabis] is legal, it's easier to monitor, and there's less room for foul play, concerning the product's quality and those who consume it. (30 year-old Arab female, Israel)
Positive economic impact	‒ Economically, [non-medical cannabis] brings in a lot of revenue for the state. (39 year-old NH Asian female, US) ‒ Recreational marijuana brings in more money and keeps people employed. (39 year-old NH Asian female, US) ‒ [Legalizing non-medical cannabis] would stimulate the economy a little bit, because I know, people would probably go out and buy that over a cigarette. (27 year-old Hispanic male, US)
Freedom of choice	‒ I think [legalizing non-medical cannabis] is good in that people can make the choice to use marijuana if they want to, as a way of having fun, or as a way of recreation. So, if you can do that, and you can still be a productive member of society and an upstanding citizen, then I think that’s okay. You should have the freedom to do that and make that choice. (30 year-old NH White male, US) ‒ Adults should be able to make their own choices regarding what they do with their time and their lives. (33 year-old NH White female, US) ‒ I’m in favor [of non-medical cannabis legalization], I think [non-medical cannabis] should be permitted… It feels to me that everyone should be able to do whatever they want with their body. (21 year-old Jewish male, Israel)
Reduced use among youth	‒ Well surely, [cannabis] is all heavily regulated and it goes out to the people who it’s supposed to go out to, and not to teenagers. And so, it’s better to go that way. Again, I think it’s better when it's regulated. When it’s being done in secret, it can be very harmful to the youth because they rely on strangers to provide them with such things, and it could affect them on the long term. (27 year-old Arab female, Israel) ‒ Perhaps if [non-medical cannabis] were to be made legal, it wouldn’t be as fun for “the kids”. Or maybe [using cannabis] still would be fun anyway, since there would be an age limitation. But it would be less challenging then, perhaps. (35 year-old Arab female, Israel)

*NH* Non-Hispanic

#### Perceptions of cannabis use

Most participants perceived cannabis use as prevalent (*“Almost the entire world smokes it.”* –27 year-old Arab female, Israel). Participants reported several concerns regarding potential risks of cannabis use. While a few commented that there is no need to shield children from cannabis use *(“Children can be aware of what is happening in the world and around them. There is no need to conceal such information from them.”* –38 year-old Jewish female, Israel), many reported concerns about using cannabis around children *(“Even if it's legal, there's still some boundaries or respect that you still should have. I would not do that in front of others and in front of children.”* –24 year-old Hispanic male, US). Some were concerned that using cannabis around children would socially normalize use among youth and expose children to cannabis byproducts (e.g., smoke). Another concern among many was use among young people, because of their developmental period and potentially leading to addiction and/or other drug use. Many also commented on cannabis’ general health risks. Relatedly, some reported concerns regarding driving under the influence of cannabis.

Regarding cannabis’ benefits, many participants commented on its potential to address physical and mental health symptoms. Some compared cannabis to opioids, alcohol, or tobacco, underscoring that they perceived cannabis to be less harmful than these other substances.

#### Perceptions of non-medical cannabis legalization

Some participants reported no substantial concerns about legalizing non-medical cannabis (“*As long as [cannabis] is well regulated, I don't have concerns.”* –39 year-old NH Asian female, US). However, some were concerned about the impact on society, including families, communities, the economy, and crime. Some also raised concerns about potential inequitable economic benefits (*“People who've been selling weed for forever and getting in trouble for it are going to be left out of the opportunity to make money now when it's legal.”* –37 year-old NH Black female, US). Another concern raised by a few participants was the price of cannabis products in a legalized market. Several also noted concerns about how cannabis products are marketed, for example, edibles appealing to young people and health claims in advertisements. Some also raised concern regarding possible insufficient regulation and quality control of legalized cannabis products.

Many commented on potential benefits of non-medical cannabis legalization (see Table 4 for example quotes). Many indicated the positive impact of decriminalization on society, including for communities disproportionately impacted by criminalization, as well as more broadly in terms of financial resources (e.g., revenue, employment, less enforcement costs). Some commented that the market might facilitate realization of cannabis’ potential medical benefits. Many also indicated that increased access to cannabis products through legal retailers (versus illegal sources) and the regulatory oversight of the legal cannabis market would reduce consumers’ overall risk in terms of products accessibility and contents. Many highlighted the importance of individual rights and freedom to choose to use. A couple suggested that legalizing cannabis use might reduce use among youth, by reducing its taboo or enforcing legal age limits.

## Discussion

Despite differences in cannabis use and related perceptions among US and Israeli adults, theory-based factors, specifically perceived risks and social norms [39], may be important targets for interventions to mitigate use-related risks among adults and youth. Such intervention efforts are critical and timely, given high cannabis use rates in the US and Israel [14], the evolving cannabis legislative context in the US [15], Israel [16], and globally [1], the potential impact of cannabis legalization on cannabis use among young people [21, 22, 28] and adults [29], and the key roles of parents and the home environment in shaping youth cannabis use [34–37].

Lower perceived risk and greater perceived social norms were associated with current use, greater use intentions, and greater intentions to use in the home or near children if legal. Furthermore, although US participants more likely reported cannabis use and favorable perceptions, Israeli participants reported greater use intentions and intentions to use in the home or among children if legalized. Particularly noteworthy is that the variables included in models tested in this study accounted for ~ 40% to ~ 63% of the variability in these outcomes, with perceived risk and social norms significantly contributing to each of the models, beyond demographic factors and cannabis use. Thus, SCT-driven interventions targeting perceived risks and social norms may help adults determine how to address cannabis use within their homes or among children and potentially mitigate use-related risks among adults and young people.

Qualitative findings suggested mixed perceptions regarding the potential impact of cannabis exposure on youth, with some participants reporting no concerns but more participants being concerned due to the potential impact on social norms and health. SCT-driven interventions targeting constructs like outcome expectancies have been shown effective in promoting rules banning tobacco smoking in private settings like homes [43–47], which reduce cigarette consumption, promote quit attempts, increase quit rates, and reduce youth tobacco use initiation [34]. These interventions provide a basis for interventions targeting cannabis-related restrictions that may lead to favorable outcomes, such as abstinence or limited use among adults and youth.

Regarding sociodemographics, males more likely used cannabis and reported greater use intentions and intentions to allow use in the home or near children if legal; bivariate analyses also indicated that not having children was associated with these cannabis-related outcomes. Identifying as a sexual minority was also associated with cannabis use. These findings align with previous studies examining correlates of use [9–13] and extend them to other cannabis-related outcomes. Interestingly, those more educated reported greater intentions to allow use in the home or near children if legal, which warrants further study.

This study also documented various perceived risks and benefits regarding cannabis use and non-medical legalization. As in previous research, qualitative results indicated perceptions that cannabis is less harmful than other substances [42, 51] and has medical benefits [56], and that legalization has various societal benefits (e.g., positive economic impact) and promotes individual rights [57]. Additionally, participants had mixed perceptions regarding whether sufficient regulation may increase safety of use and the economic impact of non-medical legalization. Moreover, participants reported additional concerns (e.g., increased crime) and benefits (e.g., increased accessibility). Collectively, these perceptions are likely influenced by various factors, including exposure to anti- and pro-legalization media [58, 59], and should be considered in future efforts to effectively communicate about changes in policy and potential risks and benefits.

Regarding use characteristics, in this sample of US and Israeli adults, US participants more likely reported lifetime (~ 50% vs. ~ 23%) and current use (22% vs. ~ 11%), and more likely obtained cannabis from legal sources (e.g., retail) and used primarily recreationally and via forms alternative to smoking (e.g., vaping, dabbing, edibles). These findings may reflect differences in access, product types, and marketing in the US resulting from legalization (as nearly half of US states have legalized non-medical cannabis [15]), proximity to legal markets, and/or shifts in social norms that have resulted from legalization [13, 28, 30–32]. Compared to 2020 data indicating past-year use prevalence of 17% among US adults and 27% among Israeli adults [14], current results indicated lower use rates among Israeli participants (likely due to different assessment timeframes, i.e., past 30-day vs. past-year) but higher use rates among US participants; this may reflect the sample’s restricted age range [18–45], as 2021 national data indicated 19.6% past-year use, with rates highest among 18–25 and 26–49 year-olds (35.4% and 24.6%) [49].

### Limitations

Despite study strengths (e.g., mixed-methods design, theory-based, cross-country), survey and interview findings may have limited generalizability, given the use of web panels and opt-in sampling to recruit the survey sample and the small sample size involved in the qualitative data collection (despite n = 84 considered a large sample for semi-structured interviews [60–63]); thus, these data are subject to selection bias and may not reflect all possible perspectives. However, our samples of survey and interview participants in each country were designed to ensure representation of the sexes, racial/ethnic groups, and tobacco use characteristics – across each subgroup (e.g., White females who reported current tobacco use vs. no use). Additionally, the cross-sectional design limits the ability to establish causal relationships between variables or assess changes over time; however, our hypotheses and analytic approach were driven by the existing literature and SCT [39]. Finally, self-report assessments of cannabis use and related characteristics introduces potential recall and social desirability biases. Cognizant of such concerns, study assessments were derived from existing published measures, neutrally worded, translated/back-translated in Israel, pilot tested for comprehension, and created to allow “refusal” to answer.

## Conclusions

The relatively high rates of cannabis use in the US and Israel [14] and rapidly shifting cannabis legislation in the US [15], Israel [16], and globally [1] underscore the need and timeliness of intervention efforts to mitigate cannabis use-related risks among adults and youth. Despite differences in cannabis use and use characteristics across countries, theory-based factors, specifically perceived risk and social norms, were shown to be relevant potential targets for interventions to mitigate cannabis use-related risks among adults and youth in the US and Israel, highlighting the importance of theory-based research across differing sociopolitical contexts.

## Data Availability

The datasets analyzed in the current study are not publicly available due to ethical reasons but are available from the corresponding author on reasonable request.

## Abbreviations

aOR: Adjusted odds ratio
CI: Confidence interval
SCT: Social Cognitive Theory
US: United States
